# Leucine-rich repeat kinase 2 controls protein kinase A activation state through phosphodiesterase 4

**DOI:** 10.1186/s12974-018-1337-8

**Published:** 2018-10-27

**Authors:** Isabella Russo, Giulietta Di Benedetto, Alice Kaganovich, Jinhui Ding, Daniela Mercatelli, Michele Morari, Mark R. Cookson, Luigi Bubacco, Elisa Greggio

**Affiliations:** 1 Department of Biology, University of Padova, Padua, Italy; 2 Present Address: Department of Molecular and Translational Medicine, University of Brescia, Viale Europa 11, 25123 Brescia, Italy; 3 Neuroscience Institute, Italian National Research Council, Padua, Italy; 4 Laboratory of Neurogenetics, National Institute on Aging, National Institutes of Health, Bethesda, MD USA; 5 Department of Medical Sciences, National Institute for Neuroscience, University of Ferrara, Ferrara, Italy

**Keywords:** LRRK2, PKA, Microglia, Neuroinflammation, Parkinson’s disease, PDE4

## Abstract

**Background:**

Evidence indicates a cross-regulation between two kinases, leucine-rich repeat kinase 2 (LRRK2) and protein kinase A (PKA). In neurons, LRRK2 negatively regulates PKA activity in spiny projecting neurons during synaptogenesis and in response to dopamine D1 receptor activation acting as an A-anchoring kinase protein (AKAP). In microglia cells, we showed that LRRK2 kinase activity negatively regulates PKA, impacting NF-κB p50 signaling and the inflammatory response. Here, we explore the molecular mechanism underlying the functional interaction between LRRK2 and PKA in microglia.

**Methods:**

To understand which step of PKA signaling is modulated by LRRK2, we used a combination of in vitro and ex vivo systems with hyperactive or inactive LRRK2 as well as different readouts of PKA signaling.

**Results:**

We confirmed that LRRK2 kinase activity acts as a negative regulator of PKA activation state in microglia. Specifically, we found that LRRK2 controls PKA by affecting phosphodiesterase 4 (PDE4) activity, modulating cAMP degradation, content, and its dependent signaling. Moreover, we showed that LRRK2 carrying the G2019S pathological mutation downregulates PKA activation causing a reduction of PKA-mediated NF-κB inhibitory signaling, which results, in turn, in increased inflammation in LRRK2 G2019S primary microglia upon α-synuclein pre-formed fibrils priming.

**Conclusions:**

Overall, our findings indicate that LRRK2 kinase activity is a key regulator of PKA signaling and suggest PDE4 as a putative LRRK2 effector in microglia. In addition, our observations suggest that LRRK2 G2019S may favor the transition of microglia toward an overactive state, which could widely contribute to the progression of the pathology in LRRK2-related PD.

## Background

Mutations in the *leucine-rich repeat kinase 2* (*LRRK2*) gene are linked to familial Parkinson’s disease (PD), and common variants increase the lifetime risk for PD [1–3]. *LRRK2* encodes a large multimeric protein characterized by an enzymatic core with GTPase and serine/threonine kinase activities and several domains surrounding these two domains that are rich in repeats involved in the assembly of signaling complexes [4]. Among all the reported LRRK2 variants, seven missense mutations, clustered within the enzymatic core of the protein, clearly segregate with disease [5], with the G2019S substitution being by far the most frequent in both familial and apparently sporadic PD cases [6]. The G2019S mutation, located in the kinase domain, augments the kinase activity of the protein as revealed by increased S1292 auto-phosphorylation [7–9] and Rabs phosphorylation [10, 11].

LRRK2 is expressed in several brain regions, including the substantia nigra pars compacta, striatum, hippocampus, cortex, and olfactory bulb [12, 13]. As well as neurons, LRRK2 is also expressed in astrocytes and microglia [14], where it has been associated with inflammatory processes related to PD [15, 16]. In this context, we recently demonstrated that microglia with LRRK2 genetic deletion or kinase inhibition exhibit a reduction of inflammation after lipopolysaccharide (LPS) or α-synuclein pre-formed fibrils (α-Syn pffs) priming. At the molecular level, we found that LRRK2 negatively regulates protein kinase A (PKA) activity, triggering an increase of PKA-mediated phosphorylation and consequent accumulation of NF-κB inhibitory subunit p50 in the nucleus, which ultimately leads to repression of NF-κB target genes [17]. A cross-talk between LRRK2 and PKA has been reported also by others [18–20]. Parisiadou and colleagues found that LRRK2 acts as a negative modulator of PKA signaling in neurons, observing that genetic deletion of LRRK2 causes increased PKA-mediated phosphorylation of glutamate receptor (GluR) 1, cAMP response element-binding protein (CREB), and cofilin resulting in abnormal synaptogenesis and transmission of striatal projection neurons [19]. Specifically, they found that LRRK2 interacts with PKA regulatory (R) IIβ subunit and that this interaction occurs between LRRK2 Ras of complex proteins (ROC) domain and PKA RIIβ dimerization domain. Moreover, they reported that PKA RIIβ is mislocalized in the dendritic spines of LRRK2 knock-out (KO) compared to wild-type (WT) neurons, leading them to hypothesize that LRRK2 regulates PKA activity by acting as an A-anchoring kinase protein (AKAP) or AKAP-like.

In its inactive form, PKA is a tetrameric enzyme composed of a R subunit dimer and two catalytic (C) subunits. In the absence of cAMP, a dimer of R subunits binds and suppresses the activity of two C subunits. Conversely, the cooperative binding of cAMP to the R subunits causes a conformational change that leads to the activation of PKA and consequent phosphorylation of its targets [21]. Typically, PKA is bound to scaffold proteins called AKAPs, which play a critical role in the compartmentalization of cAMP signaling by confining PKA to specific subcellular locations and in physical proximity to its targets [22]. PKA signaling is tightly controlled also by additional regulatory proteins that are part of the AKAP-PKA multiprotein complex, such as cAMP-degrading phosphodiesterases (PDEs), important to regulate the magnitude and duration of PKA activation, and phosphatases (PP), which dephosphorylate PKA targets to terminate the signal [23].

Building on previous observations reported by us [17] and others [19], in this study, we investigated the molecular mechanism underlying LRRK2-dependent regulation of PKA signaling in microglia. We used a combination of in vitro and ex vivo systems with hyperactive or inactive LRRK2 as well as different readouts of PKA activity, such as LRRK2-PKA RIIβ interaction and PKA RIIβ S114 phosphorylation, to evaluate the impact of LRRK2 on PKA activation. AKAP-PKA RII interaction, PKA RII phosphorylation, and regulation of cAMP content are key events that regulate the on/off state of PKA [24]. Here, we validated LRRK2 kinase activity as the negative regulator of PKA activation state in microglia cells. Moreover, we demonstrated that LRRK2 controls PKA activity through regulation of PDE4, modulating cAMP degradation, content, and its dependent signaling. We further found that LRRK2 with G2019S pathological mutation decreases PKA activity leading to a reduction of PKA-mediated NF-κB inhibitory signaling with consequent increase of inflammation in primary microglia with LRRK2 G2019S after α-Syn pffs treatment.

Taken together, our results indicate that LRRK2 kinase activity is a crucial regulator of PKA signaling in microglia and propose PDE4 as a novel LRRK2 effector protein in these cells.

## Materials and methods

### Animals

All animal procedures were carried out in strict accordance with the recommendations issued in the guidelines for the Care and Use of Laboratory Animals of the National Institutes of Health (NIH) for animals housed at NIH and for the European Community Council Directive 2010/63/UE for animals housed at the University of Padova. The protocols were approved by the Institutional Animal Care and Use Committees of the US National Institute on Aging (approval number 463-LNG-2018) and by the Ethics Committee of the University of Padova (Project ID 1041/2016-PR), respectively.

### Cell cultures

BV2 cells were cultured in RPMI-40 medium (Sigma Aldrich) supplemented with 10% fetal bovine serum (FBS), 2 mM glutamine, penicillin, and streptomycin. HEK293T cells were cultured in Dulbecco modified Eagle medium (DMEM, Life Technologies) supplemented with 10% FBS, penicillin, and streptomycin. Primary microglia cells were derived from postnatal days 1–4 (P1–4) LRRK2 wild-type and G2019S knock-in (KI) mice as recently described [17]. Specifically, cerebral cortices were mechanically dissociated in cold phosphate-buffered saline (PBS, Sigma Aldrich), then cellular suspension was allowed to settle for 5 min, and the top fraction was collected, centrifuged for 5 min at 1000*g*, and re-suspended in DMEM-F12, supplemented with 10% FBS, 2 mM glutamine, 2 mM sodium pyruvate (Sigma Aldrich), penicillin, and streptomycin. Cell suspension obtained from three brains was plated on poly-L-lysine (0.1 mg/ml, Sigma Aldrich)-coated T-75 flask. After 4 days, the medium was replaced and the mixed glial culture was maintained until day 14. At 12 days, microglia cells were isolated from the mixed culture by shaking for 4 h at 160 rpm, and the purity of the obtained culture was verified by double immunofluorescence with mouse anti-CD11b (Cell signaling #ab1211) for microglia cells and with rabbit anti-GFAP (DAKO #Z0334) for astrocytes. The amount of astrocyte contaminants was negligible.

All cells were maintained at 37 °C in a 5% CO_2_ controlled atmosphere.

### Plasmids and transfection

HEK293T cell transfections were performed using polyethylenimine (Polysciences) following the manufacturer’s recommendations. Eukaryotic expression constructs of 3xFlag-tagged LRRK2 WT and G2019S, green fluorescence protein (GFP)-tagged PKA RIIβ and GFP empty vector, generated as described previously [19, 25], were used for co-immunoprecipitation (co-IP) assays, while plasmid of GFP-tagged LRRK2 WT, generated as reported [26], was used for pull-down assays.

### Compounds and treatments

During treatments, BV2 cells were cultured in medium containing 1% FBS. LRRK2 inhibitor GSK2578215A (GSK, Tocris Bioscience) and forskolin (Sigma Aldrich) were used at 2 μM and 30 μM, respectively, for 90 min. PDE4 inhibitor rolipram (Tocris Bioscience) was used at 10 μM for 30 min. Dimethyl sulfoxide (DMSO) was used as control.

### Production and aggregation of recombinant α-Syn

Human α-Syn pffs were generated from recombinant α-Syn produced by a lipid A mutant of *Escherichia coli*, BL21(DE3), with strongly reduced endotoxicity [27]. After purification, α-Syn was incubated for 15 days to induce aggregation. α-Syn pffs were used at 25 μM for 24 h.

### Cell and brain lysates and western blotting

BV2 cells and mouse brains were solubilized as recently described [17]. Protein concentrations were determined using the BCA protein concentration assay as per manufacturer’s instructions (Thermo Scientific). Fifty micrograms of total proteins was separated by electrophoresis onto 4–20% SDS-PAGE gels and then transferred onto Immobilon-P membrane. Subsequently, membranes were incubated 1 h at room temperature (RT) with the following antibodies: rabbit anti-LRRK2 MJFF2 (1:500, Abcam #ab133474), rabbit anti-phospho S1292 LRRK2 (1:500, Abcam #ab203181), rabbit anti-p105/p50 (1:2000, Cell Signaling #13586S), rabbit anti-phospho S337 p50 (1:1000, Santa Cruz #101744), anti-flag HRP (1:20.000, Sigma Aldrich #A8592), anti-GFP (1:20.000, Roche #11814460001), mouse anti-PKA RIIβ (1:1000, BD Biosciences #610625), mouse anti-phospho S114 PKA RIIβ (1:1000, BD Biosciences #612550), goat anti-IL-1β (1:2000, R&D system #AF401NA), and mouse anti-GAPDH (1:10.000, Origene #TA150046). Then, membranes were incubated 1 h at RT with horseradish peroxidase (HRP)-conjugated secondary antibodies (Sigma Aldrich) and finally incubated with ECL western blot substrate (Thermo Scientific).

### Co-immunoprecipitation and pull-down assays

HEK293T cells were harvested at 48 h post-transfection. For co-IP assays, cells were lysed in lysis buffer (50 mM Tris pH 7.5, 1% Triton X-100, 1 mM β-glycerophosphate, 5 mM sodium pyrophosphate, 50 mM sodium orthovanadate, 0.27 M sucrose, 1 mM EDTA) and incubated with anti-Flag M2 affinity gel (Sigma Aldrich) overnight. For pull-down assays, tagged proteins were purified using GFP-trap resin (CromoTek) and then incubated for 2 h with cell lysates containing endogenous prey protein. Immuno-complexes were washed three times with lysis buffer supplemented with 0.25 M NaCl, resuspended in sample buffer, and then subjected to immunoblotting analysis.

### RNA extraction and sequencing

After shaking, isolated microglia cells were seeded for 2 days and then collected for RNA extraction, which was performed as previously described [17]. RNA quality was estimated using an Agilent 2100 Bioanalyzer RNA 6000 Nano Chip (Agilent). Samples had a mean RIN of 9. We purified RNA depleted of rRNA starting from 1 μg total RNA, then synthesized cDNA libraries using TruSeq stranded Total RNA library prep kit (Illumina, RS-122-9008) following the manufacturer’s instructions (https://support.illumina.com/content/dam/illumina-support/documents/documentation/chemistry_documentation/samplepreps_truseq/truseqstrandedtotalrna/truseq-stranded-total-rna-sample-prep-ls-euc-ltf-15031060-e.pdf). Before sequencing, cDNA libraries were quantified by digital PCR using the ddPCR Library quantification kit (Biorad). Subsequently, cDNA libraries were multiplexed with four samples per pool and 7 pM of each pool hybridized to a flow cell following cluster generation using an Illumina cluster station. Libraries were sequenced on Illumina HiSeq2500 (Illumina) to generate ~ 35 million of 100-bp single-end reads per library.

### cAMP ELISA

cAMP levels were quantified by using cAMP Elisa kit (Enzo Life Science, #ADI-900-163) according to the manufacturer’s protocol. Specifically, BV2 cells were first treated with 2 μM LRRK2 GSK inhibitor or DMSO for 90 min and then with 30 μM forskolin for 15 min to enhance cAMP contents. Five independent samples for each condition, assayed in two technical replicates, were used for the analysis.

### Statistical analysis

All quantitative data are expressed as mean ± SEM and represent at least three independent sets of experiments. Statistical significance of differences between two groups was assessed by unpaired *t* test or one-sample *t* test, while for multiple comparisons by one-way ANOVA with Tukey’s post hoc test. Data were analyzed using Prism (GraphPad).

## Results

### LRRK2^G2019S KI^ brain lysates exhibit reduced level of PKA-mediated NF-κB p50 phosphorylation

**Fig. 1:**
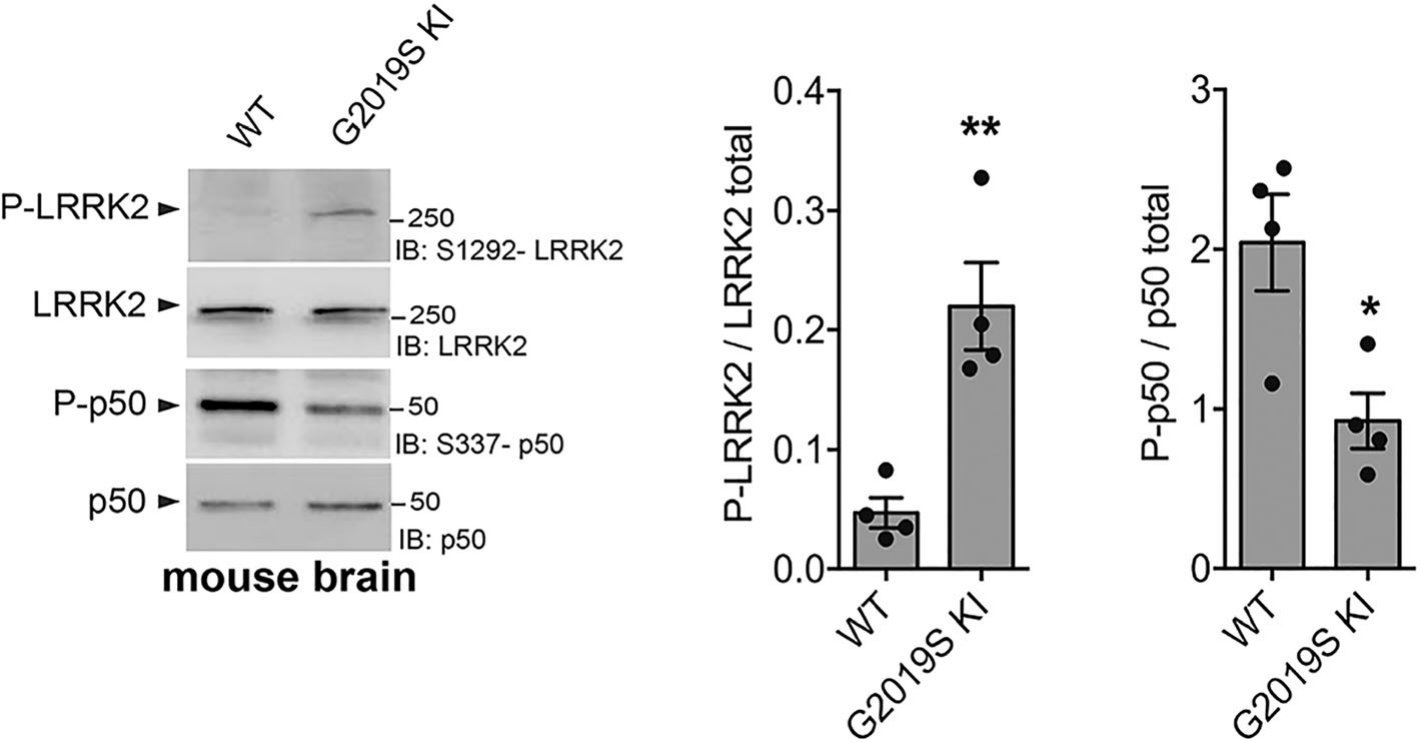
LRRK2^G2019S KI^ brain lysates exhibit reduced level of PKA-mediated NF-κB p50 phosphorylation. LRRK2 WT and LRRK2^G2019S KI^ brain lysates were subjected to immunoblotting using NF-κB P-p50, total p50, P-LRRK2, and total LRRK2 antibodies. Quantification of P-p50 subunit is normalized for total p50 protein. Quantification of P-LRRK2 is normalized for total LRRK2 protein. Data are representative of four animals (bars represent the mean ± SEM; unpaired *t* test; *p < 0.05, **p < 0.01)

We recently demonstrated that loss of LRRK2 or inhibition of its kinase activity results in increased PKA-dependent phosphorylation of NF-κB p50 inhibitory subunit at S337 [17], crucial to repress NF-κB target genes in the absence of extracellular stimulation [28]. To confirm that this regulation depends on LRRK2 kinase activity, we also explored the effect of the hyperactive LRRK2-G2019S mutation on NF-κB p50 phosphorylation. In agreement with results using LRRK2 genetic deletion or kinase activity inhibition, brain lysates from LRRK2^G2019S KI^ mice exhibited 50% reduced S337 phosphorylation compared to WT mice (Fig. 1). Moreover, as expected, LRRK2^G2019S KI^ brains display approximately fourfold enhanced kinase activity compared to WT mice, as measured by S1292 auto-phosphorylation (Fig. 1). Taken together, these results demonstrate that LRRK2 controls PKA activation state through its kinase activity.

### LRRK2 kinase activity controls LRRK2-PKA RIIβ interaction

**Fig. 2:**
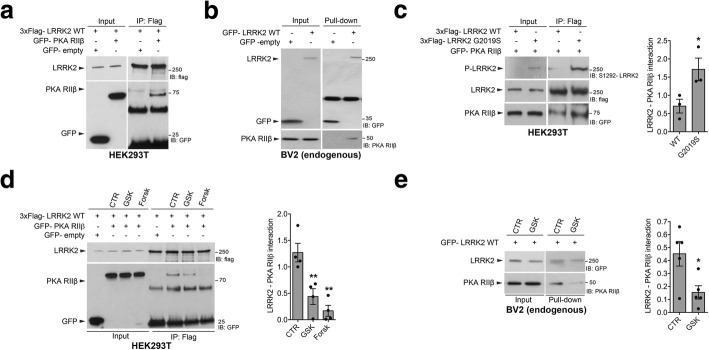
LRRK2 kinase activity regulates LRRK2-PKA RIIβ interaction. **a** Cell lysates from HEK293T co-transfected with 3xFlag LRRK2 WT and GFP-PKA RIIβ or GFP-empty vector were subjected to co-IP with anti-Flag M2 affinity gel, followed by Flag and GFP immunoblotting. **b** Pull-down assays of purified GFP-LRRK2 WT or GFP-empty vector incubated with BV2 lysates were subjected to immunoblotting with GFP and PKA RIIβ antibodies. **c** Cells lysates from HEK293T co-transfected with 3xFlag LRRK2 WT or 3xFlag LRRK2 G2019S and GPF-PKA RIIβ were subjected to co-IP with anti-Flag M2 affinity gel, followed by Flag, GFP, and P-LRRK2 immunoblotting. Quantification LRRK2- RIIβ interaction has been obtained by normalization of RIIβ for LRRK2 protein. Data are representative of three independent experiments (bars represent the mean ± SEM; unpaired *t* test; *p < 0.05). **d** Cell lysates from HEK293T co-transfected with 3xFlag LRRK2 WT and GFP-empty vector or 3xFlag LRRK2 WT and GPF- PKA RIIβ treated with GSK, forskolin (Forsk), or DMSO as control (CTR) were subjected to co-IP with anti-Flag M2 affinity gel, followed by Flag and GFP immunoblotting. Quantification LRRK2-RIIβ interaction has been obtained by normalization of RIIβ for LRRK2 protein. Data are representative of four independent experiments (bars represent the mean ± SEM; one-way ANOVA with Tukey’s post-test; **p < 0.01). **e** Pull-down assays of purified GFP-LRRK2 WT incubated with BV2 lysates previously treated with GSK or DMSO as control (CTR) were subjected to immunoblotting with GFP and PKA RIIβ antibodies. Quantification LRRK2-RIIβ interaction has been obtained by normalization of RIIβ for LRRK2 protein. Data are representative of five independent experiments (bars represent the mean ± SEM; unpaired *t* test; *p < 0.05)

LRRK2 has been reported to bind PKA RIIβ subunit and to negatively regulate PKA signaling in neurons in an AKAP-like manner [19]. Starting from these observations, we investigated whether LRRK2 also interacts with PKA RIIβ in microglia. To this end, we first validated in our hands LRRK2 interaction with PKA RIIβ by co-IP using transfected HEK293T cells. As shown in Fig. 2a, 3xFlag-LRRK2 WT interacts with GFP-PKA RIIβ but not with GFP, confirming previous findings. Next, we examined whether LRRK2 also interacts with endogenous PKA RIIβ in microglia cells. Purified GFP-LRRK2 WT, but not GFP control protein, pulls down endogenous PKA RIIβ subunit from microglial lysates (Fig. 2b), supporting the notion that this interaction occurs in different cell types.

Our results indicate that LRRK2 kinase activity influences PKA activation state. To address the underlining mechanism, we assessed whether LRRK2 kinase activity impacts LRRK2 binding to PKA RIIβ subunit. To this end, we first analyzed the interaction by co-IP upon co-transfection of 3xFlag-LRRK2 WT or 3xFlag-LRRK2 G2019S with GFP-PKA RIIβ in HEK293T cells. We found that LRRK2 with the G2019S mutation interacts more efficiently with PKA RIIβ compared to LRRK2 WT (Fig. 2c). Consistent with this idea, when we analyzed the interaction of co-transfected proteins in HEK293T cells after treatment with LRRK2 kinase inhibitor GSK, we observed a significant reduction of LRRK2-PKA RIIβ binding compared to cells treated with DMSO (Fig. 2d). Since our findings indicate that LRRK2 kinase inhibition activates PKA signaling, we next examined whether forskolin, an activator of adenylyl cyclase and indirectly of PKA, could phenocopy the interaction. Similar to GSK, forskolin treatment reduces LRRK2-PKA RIIβ binding (Fig. 2d), suggesting that when PKA is active, RIIβ has a lower affinity for LRRK2. Finally, we confirmed the reduced binding in the presence of LRRK2 kinase inhibition also by pull-down assays from microglia cells. As reported in Fig. 2, pull-down assays of GFP-LRRK2 incubated with microglial lysates previously treated with GSK inhibitor showed a reduction of interaction with endogenous PKA RIIβ compared to microglia treated with DMSO (Fig. 2e). Taken together, these results demonstrate that LRRK2 kinase activity regulates LRRK2-PKA RIIβ interaction as well as PKA activation in microglia.

### LRRK2 kinase activity affects PKA RIIβ phosphorylation at S114

**Fig. 3:**
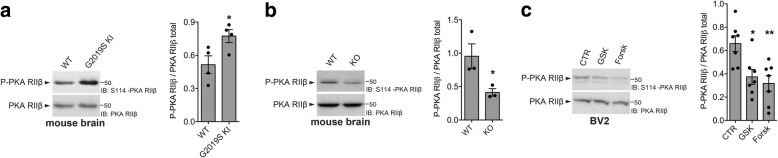
LRRK2 kinase activity affects phosphorylation of PKA RIIβ at S114. **a** LRRK2 WT and LRRK2^G2019S KI^ brain lysates were subjected to immunoblotting using P-PKA RIIβ and total PKA RIIβ antibodies. Quantification of P-PKA RIIβ is normalized for total PKA RIIβ protein. Data are representative of four animals (bars represent the mean ± SEM; unpaired *t* test; *p < 0.05). **b** LRRK2 WT and LRRK2 KO brain lysates were subjected to immunoblotting using P-PKA RIIβ and total PKA RIIβ antibodies. Quantification of P-PKA RIIβ is normalized for total PKA RIIβ protein. Data are representative of three animals (bars represent the mean ± SEM; unpaired *t* test; *p < 0.05). **c** BV2 cell lysates treated with GSK, forskolin (Forsk), and DMSO as control (CTR) were subjected to immunoblotting using P-PKA RIIβ and total PKA RIIβ antibodies. Quantification of P-PKA RIIβ is normalized for total PKA RIIβ protein. Data are representative of seven independent experiments (bars represent the mean ± SEM; one-way ANOVA with Tukey’s post-test; *p < 0.05, **p < 0.01)

Phosphorylation of RIIβ-S114, together with the regulation of cAMP content and the interaction between AKAP and PKA RIIβ, is a key event required to regulate PKA signaling [24]. Specifically, S114-RIIβ is de-phosphorylated by PPs when the C subunits are active and then re-phosphorylated by PKA catalytic subunits as a feedback regulation to inhibit the enzyme complex [24]. In the attempt of understanding which step of the PKA signaling LRRK2 affects, we explored the phosphorylation state of RIIβ using in vitro and ex vivo systems with hyperactive or inactive LRRK2. First, we evaluated RIIβ S114 phosphorylation in brain lysates from LRRK2^G2019S KI^ mice and found it is more phosphorylated compared to WT brain lysates (Fig. 3a). This result is in agreement with the observed enhancement of LRRK2-PKA RIIβ interaction and with the reduced PKA-dependent phosphorylation of p50, all indicative of an inhibition of PKA activity in the presence of LRRK2 G2019S mutation.

We subsequently examined S114-RIIβ phosphorylation in brain lysates with LRRK2 genetic deletion (Fig. 3b) and confirmed the data in microglia with LRRK2 kinase inhibition (Fig. 3c). Consistent with observations with the G2019S mutation, brain lysates from LRRK2 KO mice (Fig. 3b) and BV2 cells treated with GSK inhibitor (Fig. 3c) showed decreased levels of RIIβ phosphorylation at S114. Notably, treatment with forskolin phenocopies the effects of LRRK2 pharmacological inhibition on S114-RIIβ (Fig. 3c), further indicating that inhibition of LRRK2 activity results in increased PKA activation. These results indicate that LRRK2 kinase activity affects S114 phosphorylation of RIIβ and suggest that LRRK2 might control a downstream regulatory protein of PKA signaling.

### LRRK2 kinase activity controls cAMP levels through PDE4

**Fig. 4:**
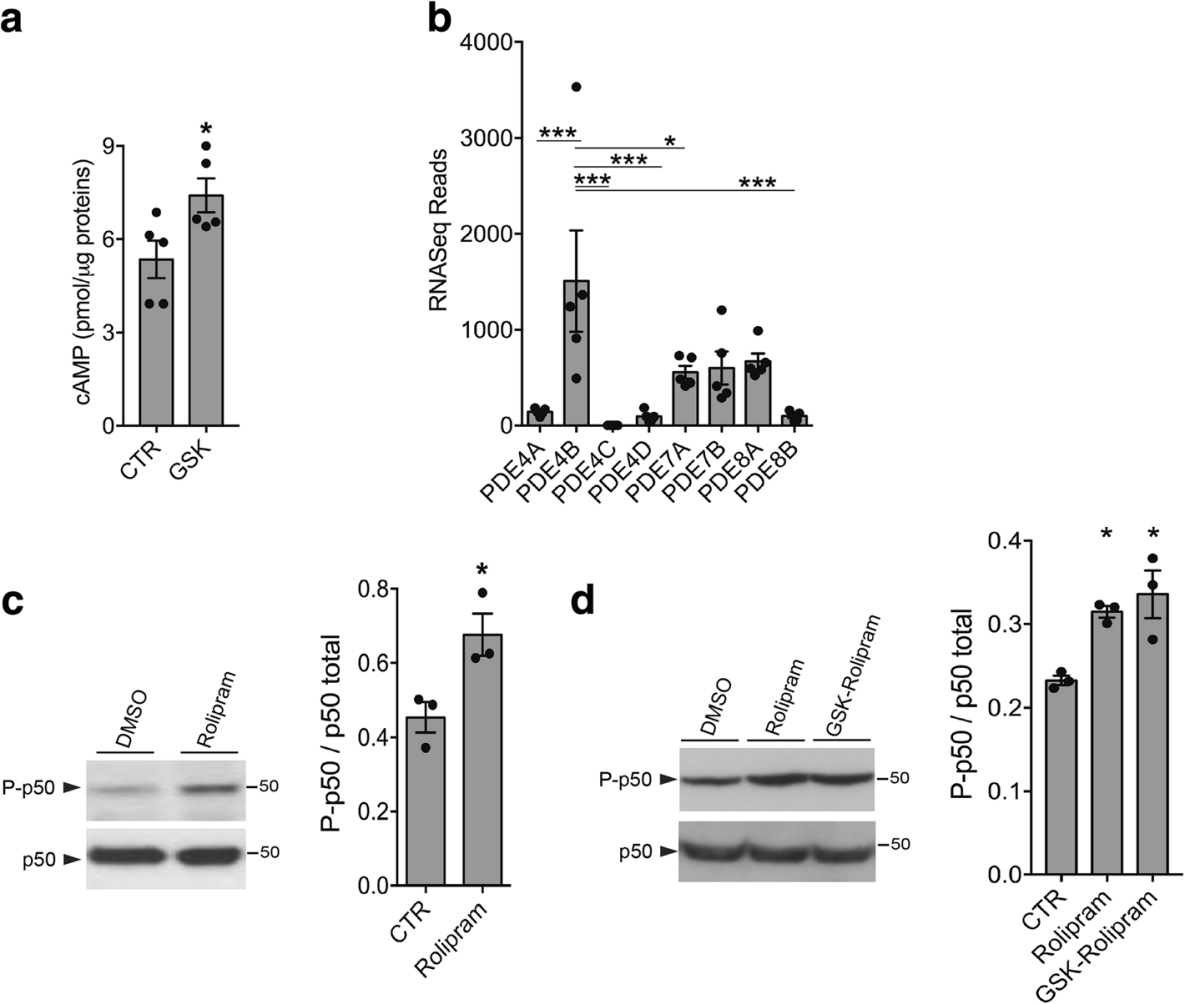
LRRK2 kinase activity controls cAMP levels through PDE4. **a** Quantification of cAMP levels in BV2 cells treated with GSK or DMSO as control (CTR). cAMP levels are normalized for total protein concentration in each sample. Data are representative of five independent experiments (bars represent the mean ± SEM; unpaired *t* test; *p < 0.05). **b** Expression of cAMP-specific PDEs generated from a RNA sequencing profile performed on WT primary microglia cells. Data are representative of five independent samples (bars represent the mean ± SEM; one-way ANOVA with Tukey’s post-test; *p < 0.05, *** *p* < 0.001). **c** BV2 cell lysates treated with rolipram or DMSO as control (CTR) were subjected to immunoblotting using NF-κB P-p50 and total p50 antibodies. Quantification of NF-κB P-p50 is normalized for total p50 protein. Data are representative of three independent experiments (bars represent the mean ± SEM; unpaired *t* test; *p < 0.05). **d** BV2 cell lysates treated with rolipram, rolipram and GSK, or DMSO as control (CTR) were subjected to immunoblotting using NF-κB P-p50 and total p50 antibodies. Quantification of NF-κB P-p50 is normalized for total p50 protein. Data are representative of three independent experiments (bars represent the mean ± SEM; one-way ANOVA with Tukey’s post-test; *p < 0.05)

The cAMP-PKA pathway is highly compartmentalized and spatiotemporally controlled through the action of PPs and PDEs signaling molecules. cAMP gradients are shaped by action of specific PDEs, which degrade cAMP and thus regulate the magnitude and duration of PKA signaling [23]. Therefore, we decided to test the hypothesis that LRRK2 may regulate PKA activation by controlling PDE-dependent cAMP degradation by quantifying total cAMP directly in microglia cells after LRRK2 kinase inhibition. Of interest, we found that cells treated with GSK exhibit increased levels of cAMP compared to cells treated with DMSO control (Fig. 4a), suggesting that LRRK2 modulates PKA activation via regulation of cAMP levels.

Cells express a defined subset of PDEs from 11 different families, three of which specifically hydrolyze cAMP (PDE4, PDE7, and PDE8) [29]. Accumulating literature indicates that among all PDEs, the B-isoform of PDE4 is the most highly expressed in immune cells, and, intriguingly, it has been proposed as a pharmacological target to reduce neuroinflammation [30]. In support of this notion, results from a RNA sequencing profile performed on WT primary microglia cells reveal that among the cAMP-specific PDEs, the PDE4B isoform is the most expressed transcript in these cells (Fig. 4b). Based on these observations, we focused our experiments on PDE4. First, we explored whether PDE4 specifically regulates PKA signaling associated with NF-κB. To this end, we analyzed PKA-dependent p50 phosphorylation in BV2 microglia cells upon treatment with the PDE4 inhibitor rolipram. We found that rolipram increases the level of p50 phosphorylation compared to cells treated with DMSO (Fig. 4c), confirming that PDE4 activity affects PKA signaling related to NF-κB p50. Next, to test whether LRRK2 acts on PKA via PDE4, we compared p50 phosphorylation of BV2 cells co-treated with PDE4 and LRRK2 inhibitors or treated with PDE4 inhibitor alone. As shown in Fig. 4d, the combined treatment of rolipram and GSK results in an increase of NF-κB p50 phosphorylation similar to that produced by rolipram alone, supporting the hypothesis that inhibition of LRRK2 kinase activity increases cAMP levels by inhibiting PDE4 activity.

### LRRK2^G2019S KI^ primary microglia exhibit increased levels of pro-inflammatory IL-1β after priming with α-Syn pffs

**Fig. 5:**
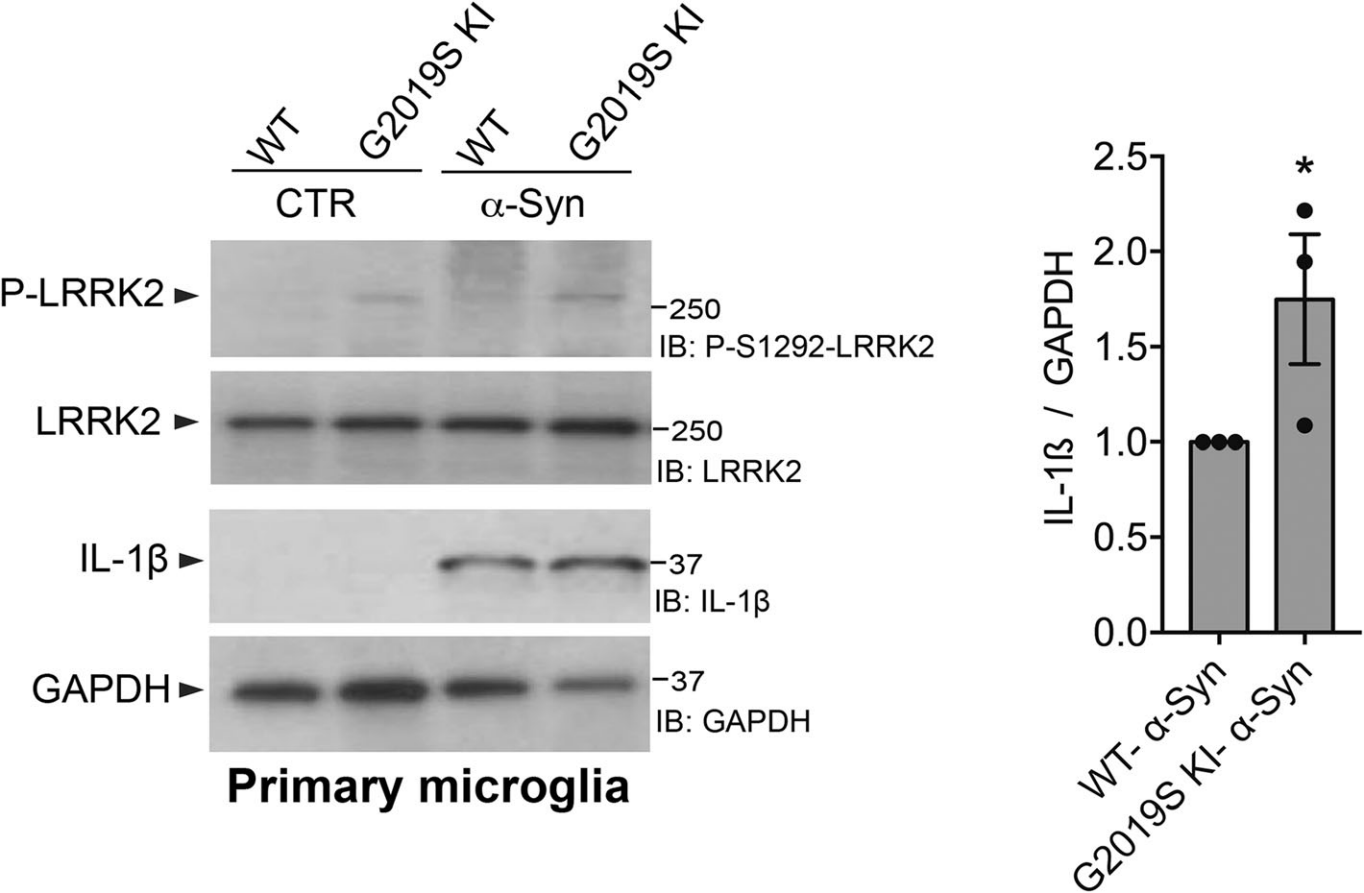
LRRK2^G2019S KI^ primary microglia exhibit increased level of pro-inflammatory IL-1β after α-Syn pffs priming. LRRK2 WT and G2019S KI microglia lysates treated with 25 μM α-Syn pffs or PBS as control (CTR) were subjected to immunoblotting using P-LRRK2, total LRRK2, IL-1β, and GAPDH antibodies. Quantification of IL-1β is normalized for GAPDH. Data are representative of three independent experiments (bars represent the mean ± SEM; one-sample *t* test; *p < 0.05)

We previously demonstrated that loss of LRRK2 or inhibition of its kinase activity causes a reduction of NF-κB-dependent transcription of inflammatory genes after LPS or α-Syn pffs priming, which is mediated by enhanced PKA activity and consequent increased NF-κB p50 inhibitory signaling [17]. In addition, here, we found that the G2019S pathological mutation reduces PKA signaling and NF-κB p50 inhibitory signaling (Figs. 1, 2, and 3). Based on this, we next asked how LRRK2 G2019S KI microglia cells would respond to an inflammatory stimulus. We treated primary microglia cells from LRRK2 WT or G2019S KI mice with α-Syn pffs and quantified pro-inflammatory IL-1β precursor (hereafter IL-1β) after 24 h of treatment. As shown in Fig. 5, α-Syn pffs induce IL-1β expression in WT microglia which is increased by about 65% in G2019S KI cells. LRRK2 S1292 auto-phosphorylation is, as expected, increased in G2019S KI microglia compared to WT cells (Fig. 5). Taken together, these findings indicate that microglia with LRRK2 G2019S exhibit an enhanced α-Syn pffs-mediated inflammation caused, at least in part, by a downregulation of cAMP/PKA signaling.

## Discussion

Accumulating evidence indicates a functional interaction between LRRK2 and PKA, although the precise molecular mechanisms of this cross-talk still need to be elucidated [31]. In this study, using in vitro and ex vivo systems with hyperactive or inactive LRRK2 and different readouts of PKA signaling, we validated LRRK2 kinase activity as the negative regulator of PKA activation. Specifically, we provided preliminary evidence that LRRK2 controls PKA activity by acting at level of PDE4, with impact on cAMP degradation, content, and its dependent signaling in microglia cells. Interestingly, we found that LRRK2 with G2019S pathological mutation downregulates PKA activity leading to an attenuation of PKA-mediated NF-κB inhibitory signaling with consequent increment of inflammation in microglia with LRRK2 G2019S KI after α-Syn pffs priming.

The available literature supports the notion that the functional interaction between LRRK2 and PKA may be bidirectional. PKA can act upstream of LRRK2 through direct phosphorylation of distinct LRRK2 serine residues [20, 32], but also LRRK2 can operate upstream of PKA and negatively regulate its activity [17, 19] with apparent different mechanisms in neurons and microglia [31]. Neuronal LRRK2 was suggested to act as an AKAP by tethering PKA signaling at specific subcellular domains independent of its kinase activity [19]. However, a very recent study by Tozzi and colleagues hypothesized that G2019S mutation is positively associated with PKA signaling in striatal medium spiny neurons [18], making the scenario even more complicated. In contrast, in microglia cells, LRRK2 kinase activity appears to be essential to regulate PKA activation/inactivation state. In support of this, we recently reported that LRRK2 kinase inhibition or genetic deletion activates PKA signaling [17]. Here we collected additional evidence that LRRK2 carrying the hyperactive G2019S mutation results in a downregulation of PKA pathway, further supporting a model where is the kinase activity of LRRK2 and not the presence of the protein itself to regulate PKA signaling in microglia cells. Overall, these observations suggest that LRRK2-dependent regulation of PKA activity might be cell-type specific.

In this study, to investigate the molecular mechanism underlying LRRK2-PKA cross-talk in microglia, we started exploring LRRK2-PKA RIIβ interaction and RIIβ phosphorylation state as readouts of PKA activation state in relation to LRRK2. Auto-phosphorylation of S114-RIIβ, or S99-RIIα, by C subunits controls the interaction between RII and C subunits [33] and the binding with AKAP [34], all key events of the activation/inactivation state of PKA. Specifically, the allosteric activation of PKA by cAMP results in the activation of C subunits, allowing the de-phosphorylation of RII dimer by PPs and its subsequent dissociation from AKAPs. In contrast, degradation of cAMP by PDEs, re-phosphorylation of RII dimer by the C subunits, and RII-AKAP re-binding cooperate to inactivate PKA signaling [24]. Here, by assessing LRRK2-PKA RIIβ interaction and RIIβ phosphorylation state, we found that LRRK2 G2019S interacts more with RIIβ compared to the WT protein in cells and brain lysates from LRRK2^G2019S KI^ mice exhibit increased phosphorylation of RIIβ compared to WT mice, suggesting that LRRK2 G2019S plays an inhibitory effect on PKA activation. In support of these results, LRRK2^G2019S KI^ mice displayed reduction of PKA-mediated NF-κB p50 phosphorylation, a well-established PKA phosphorylation target [35], whereas loss of LRRK2 or its kinase inhibition results in a decrease of LRRK2-RIIβ interaction and of S114 RIIβ phosphorylation, diagnostic of an active PKA. Taken together, these findings provide additional evidence that LRRK2 kinase activity regulates PKA by affecting S114 phosphorylation and the interaction with RIIβ subunit.

To gain more insights into the molecular mechanism of this regulation, we initially tested the hypothesis that LRRK2 modulates PKA activity through direct phosphorylation of RII subunits, but we did not find any convincing evidence from in vitro kinase assays (unpublished observations). PKA is part of a multifunctional complex composed of different signaling molecules, including PPs and PDEs, which are essential for compartmentalization and regulation of PKA activation state [36]. In particular, PDEs play a crucial role in controlling the magnitude and the duration of PKA signaling [37]. Given this key function of PDEs and the established link between PDE4 and inflammatory responses in microglia [38–41], we investigated whether LRRK2 activity affects cAMP levels in microglia. We found that cells treated with LRRK2 kinase inhibitor exhibit increased levels of cAMP compared to control cells, indicating that LRRK2 activity affects cAMP degradation. Moreover, by using phospho-S337 NF-κB p50 as readout of PKA activity, we were able to show that pharmacological manipulation of PDE4 activity impacts PKA signaling associated with NF-κB p50 phosphorylation. In addition, the combined treatment of PDE4 and LRRK2 inhibitors results in similar increase of NF-κB p50 phosphorylation compared to cells treated with rolipram alone, suggesting that LRRK2 kinase activity controls PDE4 inhibition. Future experiments will be required to elucidate the exact mechanism as to how LRRK2 regulates PDE4 activity.

Taken together, our results provide a further evidence supporting a PKA-LRRK2 axis in microglia cells, with LRRK2 kinase controlling PKA activation through PDE4; however, the molecular mechanism underlying LRRK2-PDE4 functional interaction remains to be explored. In agreement with reduced NF-κB p50 phosphorylation in the presence of hyperactive LRRK2, we found that primary microglia isolated from LRRK2^G2019S KI^ mice exhibit increased inflammation compared to WT microglia upon stimulation with α-Syn pffs. These observations suggest that LRRK2 G2019S, as well as all other pathological mutations that confer increased kinase activity, favors the transition of microglia toward a pro-inflammatory state, which, in turn, may result in an exacerbated inflammation and neurodegeneration in LRRK2-related PD patients. Supporting this hypothesis, LRRK2 G2019S carriers exhibit higher levels of peripheral NF-κB-dependent inflammatory cytokines compared to control subjects [42], and rats expressing LRRK2 G2019S display enhanced reactive microglia cells and dopaminergic neurodegeneration after intracranial injection of AAV expressing α-Syn in the substantia nigra [43].

## Conclusion

Overall, our findings indicate that LRRK2 kinase activity is a negative regulator of PKA signaling and suggest that PDE4 may be a novel LRRK2 effector protein in microglia. Future studies directed at understanding LRRK2-dependent regulation of PDE4 will offer a more defined scenario of LRRK2 biology and pathobiology in these cells. In addition, our observations suggest that LRRK2 G2019S may favor the transition of microglia toward an overactive state, which could widely contribute to the progression of the pathology in LRRK2-related PD.

## Abbreviations

AKAP: A-anchoring kinase protein
C: Catalytic
co-IP: Co-immunoprecipitation
CREB: cAMP response element-binding protein
DMEM: Dulbecco modified Eagle medium
DMSO: Dimethyl sulfoxide
FBS: Fetal bovine serum
Forsk: Forskolin
GFP: Green fluorescence protein
GluR1: Glutamate receptor R1
GSK: GSK2578215A
HRP: Horseradish peroxidase
IL-1β: Interleukin-1β
KI: Knock-in
KO: Knock-out
LPS: Lipopolysaccharide
LRRK2: Leucine-rich repeat kinase 2
NF-kB: Nuclear factor kappa-B
PBS: Phosphate-buffered saline
PD: Parkinson’s disease
PDE: Phosphodiesterase
PKA: Protein kinase A
PP: Phosphatase
RIIβ: Regulatory IIβ
ROC: Ras of complex proteins
RT: Room temperature
WT: Wild-type
α-syn pffs: α-Synuclein pre-formed fibrils
